# ﻿Review of the Palaearctic species of *Apsilocera* Bouček, 1956 (Chalcidoidea, Pteromalidae), with descriptions of the eight new species

**DOI:** 10.3897/zookeys.1215.128603

**Published:** 2024-10-17

**Authors:** Ekaterina V. Tselikh, Jaehyeon Lee, Michael Haas, Mircea-Dan Mitroiu, Deok-Seo Ku

**Affiliations:** 1 Zoological Institute, Russian Academy of Sciences, St. Petersburg 199034, Russia Zoological Institute, Russian Academy of Sciences St. Petersburg Russia; 2 Department of Plant Medicine, Gyeongsang National University, Jinju 52828, Republic of Korea Gyeongsang National University Jinju Republic of Korea; 3 Department of Entomology, State Museum of Natural History, Stuttgart, Germany State Museum of Natural History Stuttgart Germany; 4 Faculty of Biology, Alexandru Ioan Cuza University, Iasi, Romania Alexandru Ioan Cuza University Iasi Romania; 5 The Science Museum of Natural Enemies, Geochang 50147, Republic of Korea The Science Museum of Natural Enemies Geochang Republic of Korea

**Keywords:** Description, key, new record, new species, parasitoid, Pteromalinae, redescription

## Abstract

Palaearctic species of the genus *Apsilocera* Bouček, 1956 are reviewed. Twelve Palaearctic species are recognized based on females, of which eight new species are described: *Apsilocerabradburyi* Tselikh, Lee & Ku, **sp. nov.** (Republic of Korea), *A.budai* Tselikh, Lee & Ku, **sp. nov.** (Republic of Korea), *A.eleganta* Tselikh, Haas & Ku, **sp. nov.** (Republic of Korea, Sweden), *A.grandistigma* Tselikh, Lee & Ku, **sp. nov.** (Republic of Korea), *A.jejuensis* Tselikh, Lee & Ku, **sp. nov.** (Republic of Korea), *A.marina* Tselikh, Lee & Ku, **sp. nov.** (Republic of Korea), *A.totoroi* Tselikh, Haas & Ku, **sp. nov.** (Germany, Japan, Sweden), and *A.triapitzini* Tselikh, Haas & Ku, **sp. nov.** (Russia, Republic of Korea, Sweden). The female of *A.verticillata* Bouček, 1956 is described for the first time. *Apsiloceradupla* Mitroiu & Achterberg, 2013 and *A.elongata* Mitroiu & Achterberg, 2013 are recorded from the Palaearctic region for the first time. An identification key to females of all Palaearctic species of *Apsilocera* is given.

## ﻿Introduction

The pteromalid genus *Apsilocera* Bouček, 1956 (type species *Apsiloceraverticillata* Bouček, 1956) belongs to the family Pteromalidae, subfamily Pteromalinae (Burks et al. 2022), and is distributed in the Nearctic, Palaearctic, Oriental and Australian regions. Until present, it comprised nineteen species, with only *A.bramleyi* Graham, 1966 and *A.verticillata* Bouček, 1956 being found in the Palaearctic region (Bouček 1956; Graham 1966; UCD Community 2023). Another one species *A.breviscapus* Bouček, 1993 is distributed in the Nearctic region (Bouček 1993; UCD Community 2023). Thirteen other species *A.acuticristata* Mitroiu & Achterberg, 2013, *A.bicristata* Mitroiu & Achterberg, 2013, *A.brevivena* Xiao & Huang, 2001, *A.cornuta* Mitroiu & Achterberg, 2013, *A.dentata* Mitroiu & Achterberg, 2013, *A.dupla* Mitroiu & Achterberg, 2013, *A.elongata* Mitroiu & Achterberg, 2013, *A.fulvipennis* Mitroiu & Achterberg, 2013, *A.longicornis* Mitroiu & Achterberg, 2013, *A.maculata* Mitroiu & Achterberg, 2013, *A.obtusicristata* Mitroiu & Achterberg, 2013, *A.palliclava* Mitroiu & Achterberg, 2013, and *A.tuberculata* Mitroiu & Achterberg, 2013 are distributed in the Oriental region (Xiao and Huang 2001; Mitroiu and Achterberg 2013; UCD Community 2023). Three species, *A.australis* Bouček, 1988, *A.bidens* (Bouček, 1988) and *A.brevis* (Bouček, 1988) are distributed in the Australian region (UCD Community 2023).

Unfortunately, the biology of most *Apsilocera* species is unknown, but most were collected near dead trees in forests. Only *A.bramleyi* is known to parasitize small dipterans of the family Cecidomyiidae: *Cecidomyia* sp. and *Mycocecisovalis* Edwards, 1922 (Graham 1969; Ghahari and Huang 2012).

The aim of this work is to describe eight new species of *Apsilocera* from Palaearctic region. An identification key to females of all Palaearctic species of *Apsilocera* is also provided.

## ﻿Materials and methods

The specimens examined in this study are deposited in the collections of the National Institute of Biological Resources (Incheon, Republic of Korea; **NIBR**), the Science Museum of Natural Enemies (Geochang, Republic of Korea; **SMNE**), the National Museum in Prague (Prague, Czech Republic; **NMPC**), the State Museum of Natural History Stuttgart (Stuttgart, Germany; **SMNS**), the Swedish Museum of Natural History (Stockholm, Sweden; **NRM**), and the Zoological Institute of the Russian Academy of Sciences (St Petersburg, Russia; **ZISP**), the Entomological Laboratory of the Hokkaido University (Sapporo, Japan; **EIHU**), the Rijksmuseum Natuurlijke (Leiden, Netherlands; **RNHL**).

Morphological terminology, including sculpture and wing venation, follows Bouček and Rasplus (1991), Gibson (1997), and Burks et al. (2022). The flagellum consists of two anelli, six funicular segments, and the four-segmented clava. The antennal formula includes the number of segments: scapus, pedicellus, anelli, funicular segments, claval segments. The following abbreviations are used: **POL** – posterior ocellar line, the minimum distance between the posterior ocelli; **OOL** – ocello-ocular line, the minimum distance between a posterior ocellus and compound eye; **C1–C4** – claval segments; **M** – marginal vein; **S** – stigmal vein; **PM** – postmarginal vein; **F1–F6** – funicular segments; **Mt2–Mt8** – metasomal tergites (**Mt1** – petiole). The scape is measured without the radicle; the pedicel is measured in lateral view. The distance between the clypeal lower margin and the toruli is measured from the lower margins of the toruli. Eye height is measured as the maximum diameter, eye length as the minimum diameter. The mesosoma and metasoma are measured in lateral view, the latter including the ovipositor sheaths.

Specimens were examined using Olympus SZX12 and Nikon SMZ745T microscopes. Photographs were taken with a Canon EOS 70D digital camera mounted on an Olympus SZX10 microscope (IZAS, SMNE and ZISP specimens), and a Leica DFC500 digital camera attached to a Leica M205A automated research stereomicroscope (RNHL specimens). The acquired images were then processed with Helicon Focus.

## ﻿Taxonomic account

**Class Hexapoda Blainville, 1816**

**Order Hymenoptera Linnaeus, 1758**

**Family Pteromalidae Dalman, 1820**

**Subfamily Pteromalinae Ashmead, 1904**

### ﻿Genus Apsilocera

Taxon classificationAnimaliaHymenopteraPteromalidae

Bouček, 1956

3A6A3909-EB90-5C47-8AD8-25C5C17BF43D

Apsilocera Bouček, 1956: 319–121. Type species Apsiloceraverticillata Bouček, 1956, by monotypy.
Buloloa Bouček, 1988: 425–426. Type species: B.bidens Bouček, 1988, by monotypy. Synonymy by Mitroiu and Achterberg 2013: 449.
Kratinka Bouček, 1988: 428–429. Type species: K.brevis Bouček, 1988, by monotypy. Synonymy by Mitroiu and Achterberg 2013: 449, 450.
Bulolosa Bouček, 1990: 87. Replacement name for Buloloa Bouček, 1988. Synonymy by Mitroiu and Achterberg 2013: 449.

#### Diagnosis.

Head with (Figs 1, 53, 61, 69, 100, 101, 105) or without (Figs 11, 20, 27, 36, 42, 78, 85, 94, 103) ornamentation; without occipital carina. Gena usually moderately to strongly receding towards mouth, hollowed of not hollowed at mouth corner; gena lamina absent. Lower margin of clypeus with one small tooth (Fig. 91), with two small teeth (Figs 13, 27, 75), convex (Fig. 45), produced and emarginate medially (Figs 19, 35, 84), with small median projection (Fig. 105), weakly emarginate (Fig. 1), rounded (Fig. 58). Antennal formula 11264; anelli small, F1–F6 longer than broad or quadrate, with one or two rows of sensilla, antennal clava not large, symmetrical, micropilose area small. Antennal toruli situated above level of lower edges of eyes (Figs 11, 20, 27, 36, 42, 53, 69, 78, 85, 94, 100, 101, 103), sometimes on level of upper edges of eyes (Figs 1, 61); antennal protuberance and scrobes absent. Mandible formula 3:4 (Figs 11, 91) or 4:4 (Figs 35, 68).

Mesosoma short, moderately depressed or arched. Pronotum narrower than mesoscutum, with collar margin carinate (Figs 21, 28, 37, 43). Notauli incomplete (Figs 4, 10, 25, 33, 41, 49, 57, 65, 82, 90). Scutellum moderately depressed (Figs 2, 24, 89, 102, 104, 106) or arched (Figs 16, 40, 48, 64, 81, 97), without conspicuous sublateral grooves, with distinct reticulate frenal area, but without frenal groove. Metapleuron entirely smooth. Propodeum finely reticulate (Figs 49, 74), weakly alutaceous (Figs 33, 41, 57, 65, 82), smooth and shiny (Figs 4, 9, 90), plicae present; without costula and with median carina, nucha short or absent; propodeal spiracles near to front margin of sclerite. Fore wing hyaline or with infumation, with distinct speculum; M not widened proximally; M longer than S (Figs 14, 22, 30, 38, 46, 54, 62, 71, 79, 87, 95). Hind coxa dorsally bare; hind tibia with one spur.

Metasoma sessile, short, ovate (Figs 39, 55, 72, 80, 96) to acuminate (Figs 7, 15, 23, 31, 47, 63, 88), as long as or shorter than combined length of mesosoma and head. Cerci with setae subequal in length. Ovipositor not much protruding.

#### Distribution.

Nearctic, Palaearctic, Oriental and Australian regions.

### ﻿Key to Palaearctic species of *Apsilocera* based on females

**Table:** 

1	Vertex with only regular sculpture (Figs 11, 20, 27, 36, 42, 78, 85, 94, 103)	**2**
–	Vertex with various ornamentations distinctly raised above regular sculpture (Figs 1, 53, 61, 69, 100, 101, 105)	**11**
2	Clypeal margin with one small tooth (Fig. 91) or broadly convex (Fig. 45)	**3**
–	Clypeal margin with two small teeth (Figs 13, 27, 75) or produced and emarginate medially (Figs 19, 35, 84)	**5**
3	Clypeal margin broadly convex (Fig. 45). POL 1.70–1.85 × OOL. Stigma of fore wing elongate (Fig. 46)	***A.elongata* Mitroiu & Achterberg**
–	Clypeal margin with one small tooth (Fig. 91). POL 1.2–1.5 × OOL. Stigma of fore wing less elongate (Fig. 95)	**4**
4	Head in dorsal view 2.55–2.60 × as broad as long (Fig. 93) and in frontal view 1.48–1.50 × as broad as high (Fig. 94). Fore wing with M 2.10–2.20 × as long as S (Fig. 95). Antenna with scape, pedicel and F1–F6 yellowish brown, clava brown (Fig. 92)	***A.verticillata* Bouček**
–	Head in dorsal view 2.10–2.25 × as broad as long and in frontal view 1.30–1.45 × as broad as high (Fig. 103). Fore wing with M 2.4–2.5 × as long as S (Fig. 104). Antenna with scape and pedicel yellowish brown, funicle and clava brown (Fig. 104)	***A.maculata* Mitroiu & Achterberg**
5	Clypeal margin produced and emarginate medially (Figs 19, 35, 84)	**6**
–	Clypeal margin with 2 small teeth (Figs 13, 27, 75)	**9**
6	Fore wing with M 1.50 × as long as S. Eye height 4.00 × as long as malar space	***A.brevivena* Xiao & Huang**
–	Fore wing with M 1.85–2.20 × as long as S (Figs 22, 38, 87). Eye height 1.76–2.10 × as long as malar space	**7**
7	Mandible formula 4:4 (Fig. 35). F1 with many unevenly arranged sensilla (Fig. 34). Clypeal margin narrowly emarginate (Fig. 35)	***A.eleganta* Tselikh, Haas & Ku, sp. nov.**
–	Mandible formula 3:4. F1 with sparse, uniformly arranged sensilla (Figs 18, 83). Clypeal margin widely emarginate (Figs 19, 84)	**8**
8	Head and mesosoma finely reticulate (Figs 17, 20). Head in dorsal view not curved (Fig. 21). Spiracles of propodeum narrow (Fig. 17)	***A.bramleyi* Graham**
–	Head and mesosoma grossly reticulate (Figs 82, 85). Head in dorsal view curved (Fig. 86). Spiracles of propodeum not narrow (Fig. 82)	***A.triapitzini* Tselikh, Haas & Ku, sp. nov.**
9	Propodeum with reticulate sculpture (Fig. 74). Fore coxae black or dark brown (Fig. 73). Scutellum finely reticulate (Fig. 74)	***A.totoroi* Tselikh, Haas & Ku, sp. nov.**
–	Propodeum smooth (Figs 9, 25). Fore coxa yellowish brown (Fig. 24) or basally brown, apically yellowish brown (Fig. 8). Scutellum grossly reticulate (Figs 9, 25)	**10**
10	Mandible formula 4:4. Distance between antennal toruli and lower margin of clypeus 2.00–2.30 × distance between antennal toruli and median ocellus	***A.dupla* Mitroiu & Achterberg**
–	Mandible formula 3:4 (Fig. 11). Distance between antennal toruli and lower margin of clypeus 3.00–3.20 × distance between antennal toruli and median ocellus	***A.bradburyi* Tselikh, Lee & Ku, sp. nov.**
11	Vertex normal, not high, with a row of teeth originating near eye and ending near posterior ocellus (Fig. 69, 105). Head in frontal view 1.40–1.60 × as broad as high (Fig. 69, 105)	**12**
–	Vertex high (Figs 1, 53, 61, 100, 101), with one (Figs 1, 61, 100) or two (Fig. 53, 101) crests or elevations between posterior ocelli and anterior ocellus. Head in frontal view 1.00–1.19 × as broad as high (Figs 1, 53, 61, 100, 101)	**13**
12	Clypeal margin with small median projection (Fig. 105). Eye height 1.14 × eye length (Fig. 106) and 2.00 × as long as malar space	***A.tuberculata* Mitroiu & Achterberg**
–	Clypeal margin with two teeth (Fig. 66). Eye height 1.31–1.35 × eye length and 1.60–1.67 × as long as malar space	***A.marina* Tselikh, Lee & Ku, sp. nov.**
13	Vertex with two distinct crests not composed of small sharp teeth, leaving a depression in middle (Fig. 53, 101)	**14**
–	Vertex with one continuous crest composed of small sharp teeth, leaving no depression in middle (Figs 1, 61, 100)	**15**
14	POL 1.70–1.85 × OOL. Antenna with scape 1.37 × as long as eye length. Propodeum smooth. Fore wing with M 1.93 × as long as S, stigma small (Fig. 102)	***A.bicristata* Mitroiu & Achterberg**
–	POL 1.45 × OOL. Antenna with scape 1.03 × as long as eye length. Propodeum finely reticulate (Fig. 49). Fore wing with M 1.50 × as long as S, stigma enlarged (Fig. 54)	***A.grandistigma* Tselikh, Lee & Ku, sp. nov.**
15	Crest of vertex moderately high (Fig. 99, 100). Distal tooth of mandible blunt. POL 1.08 × OOL	***A.acuticristata* Mitroiu & Achterberg**
–	Crest of vertex very high (Figs 1, 61). Distal tooth of mandible not blunt (Figs 1, 61). POL 0.77–0.85 × OOL	**16**
16	Crest height 0.80 × eye length (Fig. 1). Clypeal margin weakly emarginate (Fig. 1). Distance between antennal toruli and lower margin of clypeus 3.40 × distance between antennal toruli and median ocellus. Head grossly reticulate (Fig. 1)	***A.budai* Tselikh, Lee & Ku, sp. nov.**
–	Crest height 0.50 × eye length (Fig. 61). Clypeal margin rounded (Fig. 58). Distance between antennal toruli and lower margin of clypeus 4.00–4.15 × distance between antennal toruli and median ocellus. Head finely reticulate (Fig. 61)	***A.jejuensis* Tselikh, Lee & Ku, sp. nov.**

### ﻿ Apsilocera bradburyi

Taxon classificationAnimaliaHymenopteraPteromalidae

Tselikh, Lee & Ku sp. nov.

95609959-4DCC-53EC-AFE9-EC466F62F2EB

https://zoobank.org/592086BF-94C3-489C-9B0D-3B04D9C4CA6A

Figs 8–15

#### Type material.

***Holotype*** • female, Republic of Korea: “Gyeongsangbuk-do, Andong-si, Bukhu-myeon, Dahyeon-ri, Malaise trap, coll. Kwon Gi-myon” (NIBR). ***Paratypes*** • 3 females, “Jeju-do, Jeju-si, Geumak-ri, Hanllim-eup, 16.VI–14.VII.2021, 16.VII–13.X.2021, Malaise trap, coll. Y.H. Park, M.H. Kim, D.H. Park, J.Y. Kim (1 female in ZISP, 2 females in SMNE) • 1 female, “Jeju-do, Jeju-si, Jocheon-eup, Gyorae Natual Recreate, 33°26'28"N, 126°39'53"E, 13.VII.2023, coll. E. Tselikh” (ZISP).

#### Description.

**Female.** Body length 1.50–1.70 mm; fore wing length 1.40–1.50 mm.

***Coloration*.** Head black; antenna with scape, pedicel, and anelli yellow, F1–F6 yellowish brown, clava brown. Mesosoma black, but propodeum dorsally dark blue with metallic diffuse luster; fore coxa basally brown, apically yellowish brown, mid and hind coxae yellowish brown, all femora yellowish brown, tibiae and tarsi yellow. Fore wing hyaline, venation yellowish brown. Metasoma dorsally brown, laterally and ventrally yellowish brown; ovipositor sheaths brown.

***Sculpture*.** Head reticulate; clypeus radially striate. Mesosoma reticulate; propodeum smooth. Metasoma weakly alutaceous and shiny.

***Head*.** Head in dorsal view 2.50–2.53 × as broad as long and 1.31–1.36 × as broad as mesoscutum; in frontal view 1.42–1.45 × as broad as high. Vertex with regular sculpture. POL 1.13–1.25 × as long as OOL. Eye height 1.29–1.33 × eye length and 2.00–2.10 × as long as malar space. Distance between antennal toruli and lower margin of clypeus 2.90–3.15 × distance between antennal toruli and median ocellus. Antenna with scape 0.90–0.95 × as long as eye height and 1.09–1.16 × as long as eye length; pedicel 1.57–1.66 × as long as broad; combined length of pedicel and flagellum 1.04–1.10 × breadth of head; F1–F6 longer than broad, with 1 row of sensilla; clava 2.88–3.16 × as long as broad, with small micropilose area on each C3 and C4. Clypeal margin with two small teeth. Mandible formula 3:4.

***Mesosoma*.** Mesosoma 1.22–1.31 × as long as broad. Scutellum moderately arched, 0.82 × as long as broad, frenal area indistinct. Propodeum 0.37–0.41 × as long as scutellum; nucha short. Fore wing 2.13–2.22 × as long as its maximum width; basal cell with several hairs near basal vein; basal vein pilose; speculum partly closed below; M 0.85–0.87 × as long as PM and 2.17–2.20 × as long as S; stigma small.

***Metasoma*.** Metasoma 1.73–2.03 × as long as broad, 1.50–1.55 × as long as mesosoma. Petiole strongly transverse. Ovipositor sheath projecting slightly beyond apex of metasoma.

**Male.** Unknown.

#### Etymology.

The species is named in honor of the famous writer Ray Douglas Bradbury.

#### Distribution.

Korean Peninsula.

#### Comments.

*Apsilocerabradburyi* sp. nov. belongs to a group of species that have a vertex with regular sculpture. This species is very similar to *A.dupla*; the differences between these species are given in the key.

### ﻿ Apsilocera bramleyi

Taxon classificationAnimaliaHymenopteraPteromalidae

Graham, 1966

AA5D2347-534A-584A-9CEC-8328DB454B2A

Figs 16–23

Apsilocera bramleyi Graham, 1966: 301–304. Holotype female (HDOU, not examined).

#### Additional material examined.

Japan • 1 female, “Yokohama, Kanagawa Pref., 11.VI.2002, coll. K. Kubo” (EIHU). Russia • 1 female, “Krasnodar Reg., Sochi, Lazarevskoe, VIZR, 21.VII.1975, coll. V. Triapitzin” (ZISP). Germany • 1 female (SMNS_Hym_Pte_001795), “D, Sachsen, Lkr. Bautzen, Luppa, 51.278N, 14.403183E, 164 m, Sweep net, coll. L. Krogmann, T. Kothe”, Sample ID: SMNS_38858 (SMNS). Sweden • 1 female (SMNS_Hym_Pte_006119), “Sweden, Mörbylånga, Lilla Vickleby Lunds NR (#115/2014), old oak forest, 27.VI–30.VII.2014, Malaise trap, 56.567331N, 16.441516E, coll. M. Jaschhof, C. Jaschhof”, Sample ID: SMNS_48289 (SMNS); • 1 female (SMNS_Hym_Pte_005876), “Sweden, Mörbylånga, Lilla Vickleby Lunds NR (#137/2014), old oak forest, 31.VII–29.VIII.2014, Malaise trap, 56.567331N, 16.441516E, coll. M. Jaschhof, C. Jaschhof”, Sample ID: SMNS_48046 (SMNS).

#### Biology.

Primary parasitoid of *Cecidomyia* sp. and *Mycocecisovalis* Edwards, 1922 (Diptera, Cecidomyiidae) (Graham 1969; Ghahari and Huang 2012).

#### Distribution.

France, Germany, Iran, Japan, Russia, Serbia, Sweden, United Kingdom.

#### Comments.

*Apsilocerabramleyi* belongs to a group of species that have a vertex with regular sculpture. This species is very similar to *A.triapitzini* sp. nov.; the differences between these species are given in the key.

### ﻿ Apsilocera budai

Taxon classificationAnimaliaHymenopteraPteromalidae

Tselikh, Lee & Ku sp. nov.

EEC75457-D672-5DA7-8C3F-8C8B5465E869

https://zoobank.org/2C0500D4-3403-4AF9-B5CA-1A0C06F2110E

Figs 1–7

#### Type material.

***Holotype*** • female, Republic of Korea: “Gyeongsangnam-do, Goseong-gun, Hail-myeon, Suyang-ri, 34°58'34.8"N, 128°12'08.3"E, 18.VI.2022, coll. E. Tselikh” (NIBR).

#### Description.

**Female.** Body length 1.40 mm; fore wing length 1.30 mm.

***Coloration*.** Head black, in frontal view dark green with metallic diffuse luster; antenna yellowish brown. Mesosoma black, but propodeum dorsally dark blue-green with metallic diffuse luster; all coxa, all femora and tibiae yellowish brown, all tarsi yellow. Fore wing hyaline, venation yellowish brown. Metasoma dark brown with metallic green, diffuse coppery luster; ovipositor sheaths dark brown.

***Sculpture*.** Head grossly reticulate; clypeus radially striate, but near clypeal margin smooth. Mesosoma grossly reticulate; propodeum smooth. Metasoma weakly alutaceous and shiny.

***Head*.** Head in dorsal view 2.39 × as broad as long and 1.27 × as broad as mesoscutum; in frontal view 1.00 × as broad as high. Vertex with one continuous crest composed of small sharp teeth, leaving no depression in middle; crest height 0.80 × eye length. POL 0.77 × as long as OOL. Eye height 1.31 × eye length and 1.79 × as long as malar space. Distance between antennal toruli and lower margin of clypeus 3.46 × distance between antennal toruli and median ocellus. Antenna with scape 1.18 × as long as eye height and 1.54 × as long as eye length; pedicel 1.31 × as long as broad; combined length of pedicel and flagellum 1.26 × breadth of head; F1–F6 longer than broad, with 1 row of sensilla; clava 2.63 × as long as broad, with small micropilose area on each C3 and C4. Clypeal margin produced and weakly emarginate medially.

***Mesosoma*.** Mesosoma 1.30 × as long as broad. Scutellum moderately depressed, 0.96 × as long as broad, frenal area indistinct. Propodeum 0.38 × as long as scutellum; nucha short. Fore wing 2.24 × as long as its maximum width; basal cell with several hairs near submarginal vein; basal vein pilose; speculum partly closed below; M 0.98 × as long as PM and 2.17 × as long as S; stigma small.

**Figures 1–7.**
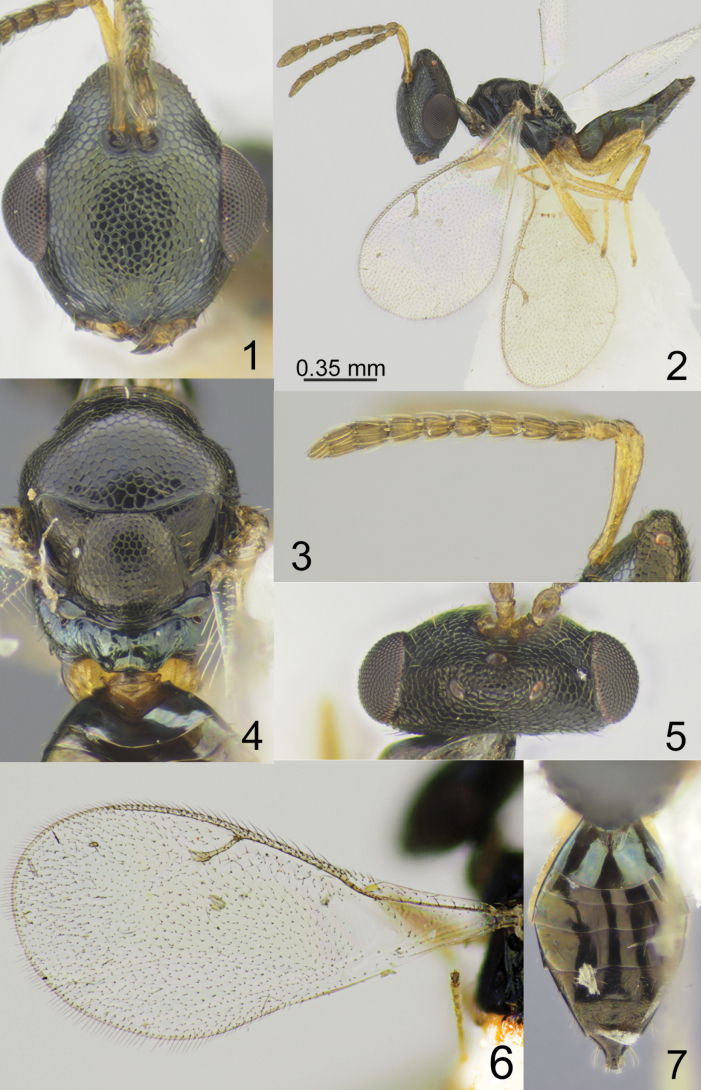
*Apsilocerabudai* sp. nov., female, holotype **1** head, frontal view **2** habitus, lateral view **3** antenna **4** mesosoma, dorsal view **5** head, dorsal view **6** fore wing **7** metasoma, dorsal view.

**Figures 8–15.**
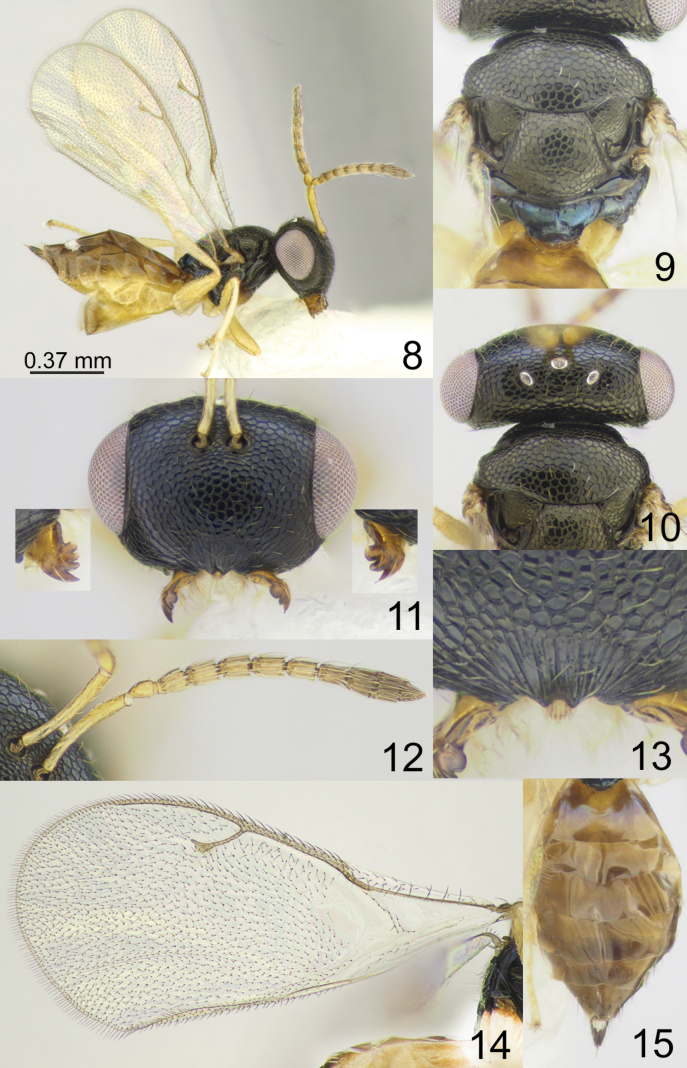
*Apsilocerabradburyi* sp. nov., female, holotype **8** habitus, lateral view **9** mesosoma, dorsal view **10** head, pronotum and mesoscutum, dorsal view **11** head, mandible, frontal view **12** antenna **13** clypeus **14** fore wing **15** metasoma, dorsal view.

**Figures 16–23.**
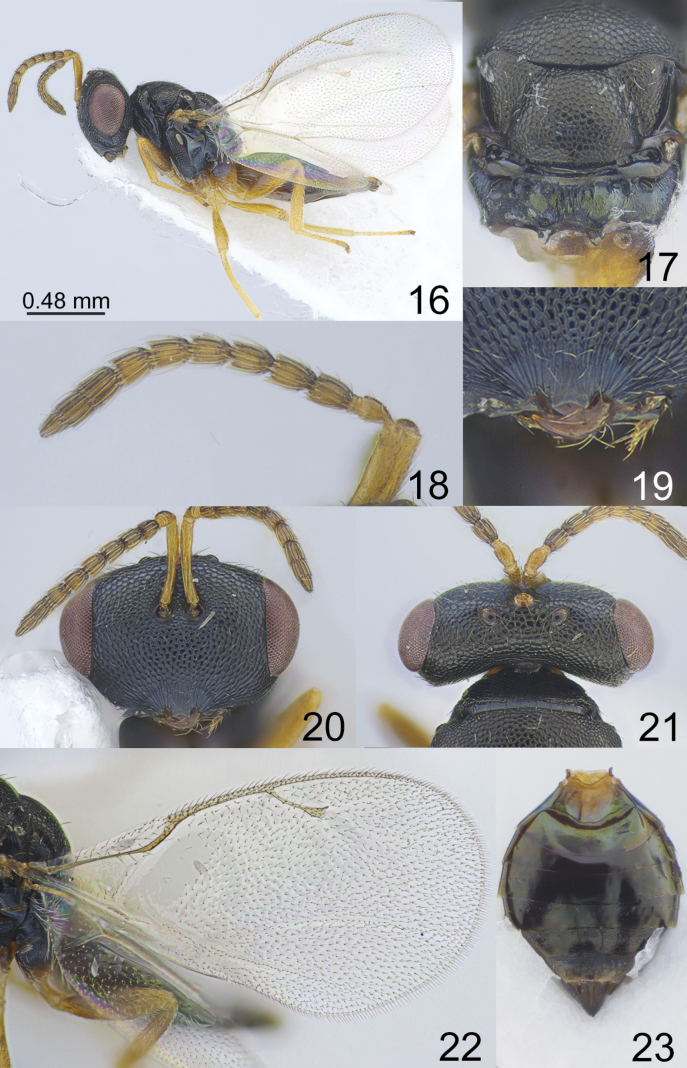
*Apsilocerabramleyi* Graham, 1966, female, not type **16** habitus, lateral view **17** scutellum and propodeum, dorsal view **18** antenna **19** clypeus **20** head, frontal view **21** head and pronotum, dorsal view **22** fore wing **23** metasoma, dorsal view.

***Metasoma*.** Metasoma 1.63 × as long as broad, 1.19 × as long as mesosoma. Petiole strongly transverse. Ovipositor sheath projecting slightly beyond apex of metasoma.

**Male.** Unknown.

#### Etymology.

The species is named in honor of the large golden statue of Buddha in the type locality.

#### Distribution.

Korean Peninsula.

#### Comments.

*Apsilocerabudai* sp. nov. belongs to a group of species that have a vertex with one continuous crest composed of small sharp teeth. This species is very similar to *A.jejuensis* sp. nov.; the differences between these species are given in the key.

### ﻿ Apsilocera dupla

Taxon classificationAnimaliaHymenopteraPteromalidae

Mitroiu & Achterberg, 2013

E70B59ED-7B56-54D0-9663-3C424C9E6828

Figs 24–31

Apsilocera dupla Mitroiu & Achterberg, 2013: 451, 461–462. Holotype female (RNHL, examined).

#### Type material.

***Holotype*** • female, Vietnam: “N. VIETNAM: Hoa Binh, Hang Kia Pà Cò N.R., 1329 m, 20°44'36"N, 104°53'45"E, 2.III–15.IV.2011, Mal. tr. 5, C. v. Achterberg, RMNH’11” (RMNH).

#### Additional material examined.

Republic of Korea • 2 females, 1 male, “Gyeongsangbuk-do, Yeongyang-gun, Irwol-myeon, Mt. Ilwolsan, 36°48'29"N, 129°05'25"E, 7.VII.2015, coll. E. Tselikh” (ZISP) • 1 female, “Jeollanam-do, Goheung-gun, Geumsan-myeon, Eojeon-ri, 3.VIII–16.VIII.2020, Malaise trap, coll. D.S. Ku, J.H. Lee” (NIBR) • 2 females, “Gyeongsangnam-do, Jinju-si, Ilbanseong-myeon, Changchon-ri, 4.VI–20.VI.2022, 25.VI–16.VII.2022, Malaise trap, coll. An Tae-Ho” (SMNE) • 5 females, “Gyeongsangnam-do, Geoje-si, Hacheong-myeon, Eoon-ri, 34°59'24.6"N, 128°38'27"E, 2.VII.2023, coll. E. Tselikh (ZISP).

#### Distribution.

Indonesia, Vietnam, Korean Peninsula.

#### Comments.

*Apsiloceradupla* belongs to a group of species that have a vertex with regular sculpture. This species is very similar to *Apsilocerabradburyi* sp. nov.; the differences between these species are given in the key.

### ﻿ Apsilocera eleganta

Taxon classificationAnimaliaHymenopteraPteromalidae

Tselikh, Haas & Ku sp. nov.

C44702AE-A72B-5DB8-BB0F-52B3A1EF98B1

https://zoobank.org/FEFF5EEE-761D-4D06-890B-DB485B647AC2

Figs 32–39

#### Type material.

***Holotype*** • female, Republic of Korea: “Gyeonggi-do, Pocheon-si, Soheul-eup, Jikdong-ri, Malaise trap, 30.X.2009, coll. S.Y. Park” (NIBR). ***Paratypes*** • 1 female (NHRS-HEVA000022836), “Sweden Öl, Mörbylånga kommun, Västerstads almlunds naturreservat., Old elm forest (3002 Trap ID, 3053 Event ID), 10.VI–9.VII.2014, Malaise trap, 56.427648N, 16.421110E, leg. Swedish Malaise Trap Project”, Sample ID: SMNS_50686 (NRM) • 1 female (SMNS_Hym_T00802), “Sweden Sm, Nybro kommun, Alsterbro/Alsterån, Mixed forest (1008 Trap ID, 2000 Event ID), 5.IX–13.X.2006, Malaise trap, 56.936536N, 15.920167E, leg. Swedish Malaise Trap Project”, Sample ID: SMNS_115563 (SMNS); • 1 female (NHRS-HEVA000022837), “Sweden Sm, Nybro kommun, Alsterbro/Alsterån, Mixed forest (1008 Trap ID, 1746 Event ID), 8.VIII–19.VIII.2006, Malaise trap, 56.936536N, 15.920167E, leg. Swedish Malaise Trap Project” (NRM).

**Figures 24–31.**
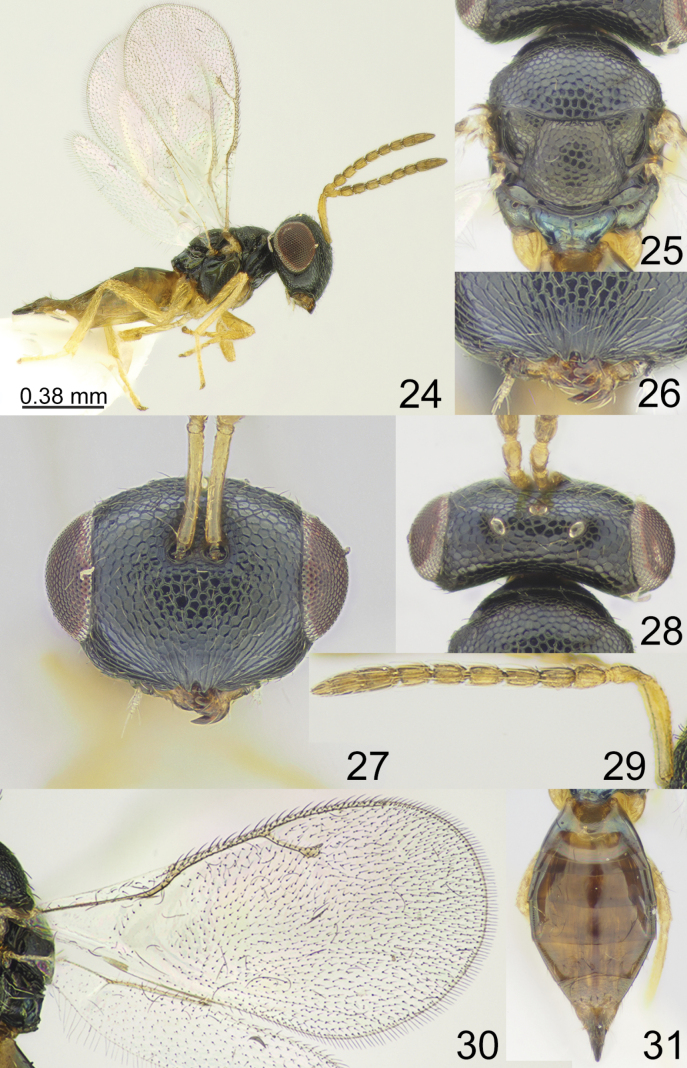
*Apsiloceradupla* Mitroiu & Achterberg, 2013, female, not type **24** habitus, lateral view **25** mesosoma, dorsal view **26** clypeus **27** head, frontal view **28** head and pronotum, dorsal view **29** antenna **30** fore wing **31** metasoma, dorsal view.

**Figures 32–39.**
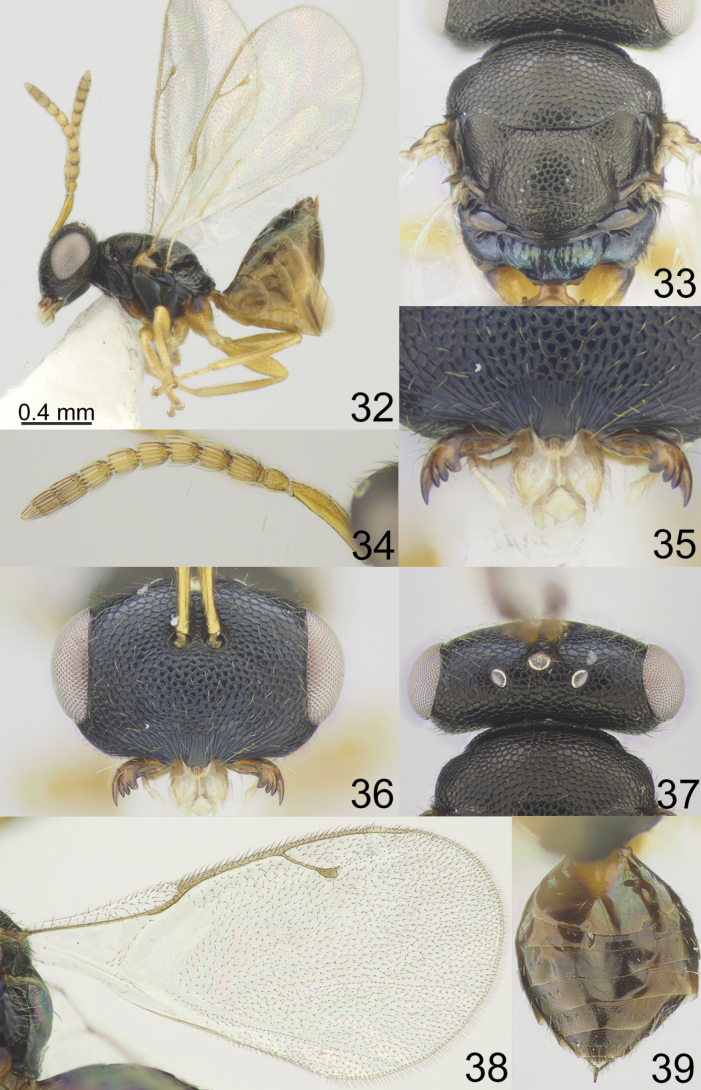
*Apsiloceraeleganta* sp. nov., female, holotype **32** habitus, lateral view **33** mesosoma, dorsal view **34** antenna **35** clypeus **36** head, frontal view **37** head and pronotum, dorsal view **38** fore wing **39** metasoma, dorsal view.

#### Description.

**Female.** Body length 1.60–2.82 mm; fore wing length 1.65–2.11 mm.

***Coloration*.** Head black; antenna yellowish brown. Mesosoma black, but propodeum dorsally dark blue with metallic diffuse luster; all coxae basally metallic blue, apically yellowish brown; all femora, tibiae and tarsi yellowish brown. Fore wing hyaline, venation yellowish brown. Metasoma dorsally brown, laterally and ventrally yellowish brown; ovipositor sheaths yellowish brown.

**Figures 89–96.**
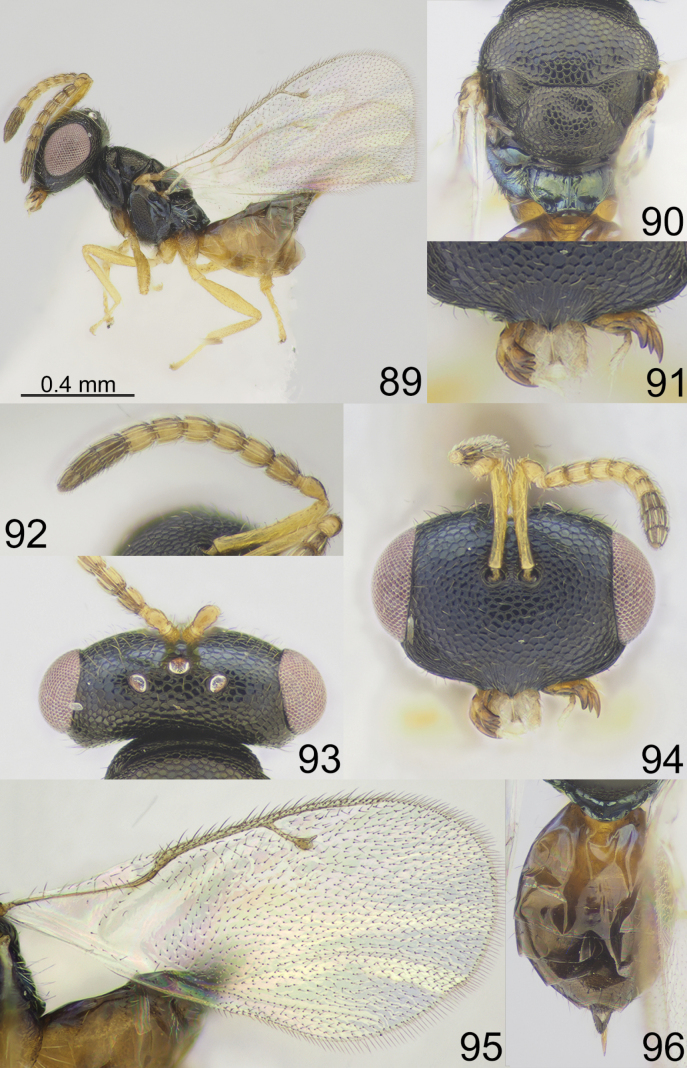
*Apsiloceraverticillata* Bouček, 1956, female, not type **89** habitus, lateral view **90** mesosoma, dorsal view **91** clypeus and mandible **92** antenna **93** head and pronotum, dorsal view **94** head, frontal view **95** fore wing **96** metasoma, dorsal view.

**Figures 97–106.**
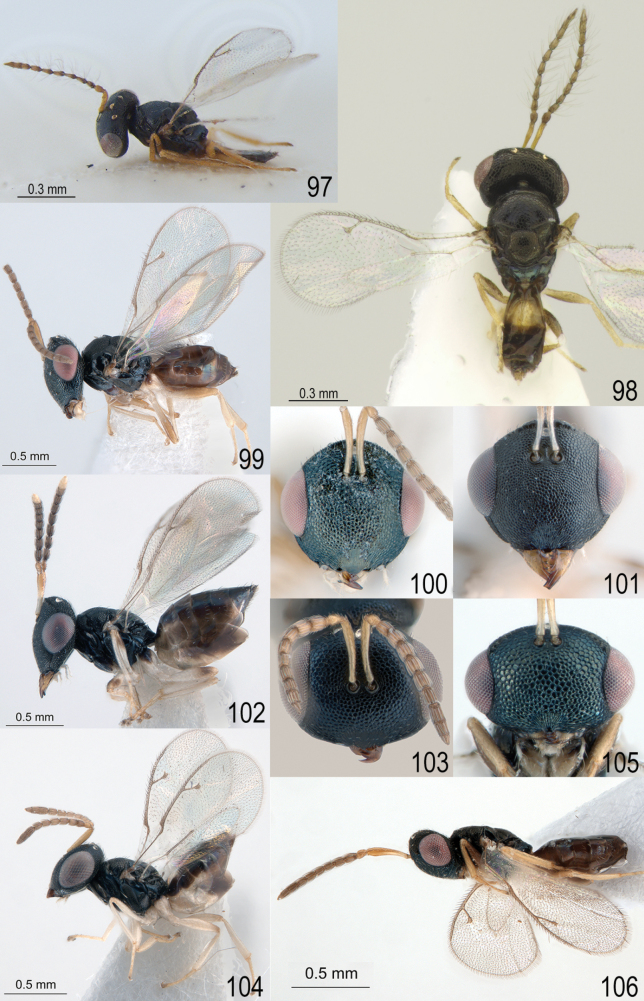
**97***Apsiloceraverticillata* Bouček, 1956, male, holotype habitus, lateral view **98***Apsiloceratriapitzini* sp. nov., male, paratype habitus, dorsal view **99, 100***Apsiloceraacuticristata* Mitroiu & Achterberg, 2013, female, holotype **99** habitus, lateral view **100** head, frontal view **101, 102***Apsilocerabicristata* Mitroiu & Achterberg, 2013, female, holotype **101** head, frontal view **102** habitus, lateral view **103, 104***Apsiloceramaculata* Mitroiu & Achterberg, 2013, female, holotype **103** head, frontal view **104** habitus, lateral view **105, 106***Apsiloceratuberculata* Mitroiu & Achterberg, 2013, female, holotype **105** head, frontal view **106** habitus, lateral view.

***Sculpture*.** Head reticulate; clypeus radially striate, but near clypeal margin smooth. Mesosoma reticulate; propodeum alutaceous. Metasoma weakly alutaceous and shiny.

***Head*.** Head in dorsal view 2.43–2.65 × as broad as long and 1.27–1.32 × as broad as mesoscutum; in frontal view 1.49–1.58 × as broad as high. Vertex with regular sculpture. POL 1.14–1.19 × as long as OOL. Eye height 1.37–1.40 × eye length and 1.76–2.00 × as long as malar space. Distance between antennal toruli and lower margin of clypeus 2.50–2.61 × distance between antennal toruli and median ocellus. Antenna with scape 0.92–0.95 × as long as eye height and 1.28–1.31 × as long as eye length; pedicel 1.50–1.66 × as long as broad; combined length of pedicel and flagellum 0.94–0.98 × breadth of head; F1, F2 longer than broad, F3–F6 subquadrate, with single row of sensilla; clava 2.85–3.18 × as long as broad, with small micropilose area on each C3 and C4. Clypeal margin produced and emarginate medially. Mandible formula 4:4.

***Mesosoma*.** Mesosoma 1.23–1.34 × as long as broad. Scutellum moderately depressed, 0.78 × as long as broad, frenal area indistinct. Propodeum 0.37–0.43 × as long as scutellum. Fore wing 1.94–2.01 × as long as its maximum width; basal cell with several hairs; basal vein pilose; speculum partly closed below; M 0.72–0.89 × as long as PM and 1.73–2.06 × as long as S; stigma small.

***Metasoma*.** Metasoma 1.19–1.50 × as long as broad and 1.05–1.20 × as long as mesosoma.

**Male.** Unknown.

#### Etymology.

From the Latin *elegans*, referring to the elegant habitus of this species.

#### Distribution.

Korean Peninsula, Sweden.

#### Comments.

*Apsiloceraeleganta* sp. nov. belongs to a group of species that have a vertex with regular sculpture. This species is very similar to *A.bramleyi* and *A.triapitzini* sp. nov.; the differences between these species are given in the key.

### ﻿ Apsilocera elongata

Taxon classificationAnimaliaHymenopteraPteromalidae

Mitroiu & Achterberg, 2013

9876D516-5370-58D7-B254-D75A952B2651

Figs 40–47

Apsilocera elongata Mitroiu & Achterberg, 2013: 451, 462. Holotype female (RNHL, examined).

#### Type material.

***Holotype*** • female, Vietnam: “N. VIETNAM: Ninh Binh, Cuc Phuong N.P., nr centre (I), c 225 m, 20.XII.1999–10.II.2000, Mai Phu Quy, RMNH’00” (RMNH).

**Figures 40–47.**
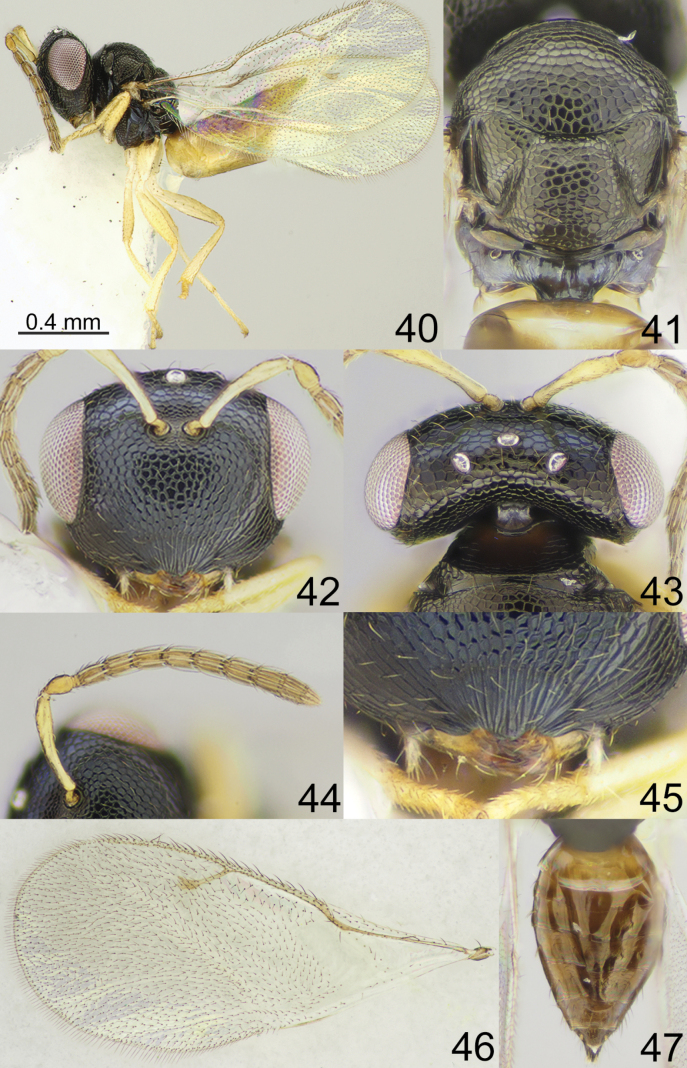
*Apsiloceraelongata* Mitroiu & Achterberg, 2013, female, not type **40** habitus, lateral view **41** mesosoma, dorsal view **42** head, frontal view **43** head and pronotum, dorsal view **44** antenna **45** clypeus **46** fore wing **47** metasoma, dorsal view.

#### Additional material examined.

Republic of Korea • 1 female, “Gyeongsangbuk-do, Ulijn-gun, Geumgangsong-myeon, Sogwang-ri, Malaise trap, 17.VIII.2016, coll. H.K. Lee” (NIBR).

#### Distribution.

Korean Peninsula, Vietnam.

#### Comments.

*Apsiloceraelongata* sp. nov. belongs to a group of species that have a vertex with regular sculpture. This species is very similar to *A.verticillata* and *A.maculata*; the differences between these species are given in the key.

### ﻿ Apsilocera grandistigma

Taxon classificationAnimaliaHymenopteraPteromalidae

Tselikh, Lee & Ku sp. nov.

7D58F481-1596-5515-BB05-0BCD8FD65399

https://zoobank.org/844D223D-603C-4F56-8131-74805087F4B1

Figs 48–55

#### Type material.

***Holotype*** • female, Republic of Korea: “Gyeonggi-do, Pocheon-si, Soheul-eup, 37°45'01.6"N, 127°08'34.9"E, 24.V–12.VI.2017, coll. Kim, Kim, Nam” (NIBR).

#### Description.

**Female.** Body length 1.40 mm; fore wing length 1.40 mm.

***Coloration*.** Head black; antenna with scape, pedicel, and anelli yellowish brown, F1–F6 and clava brown. Mesosoma black; fore and mid coxa black, hind coxae basally black, apically yellowish brown, all femora yellowish brown, all tibiae and tarsi yellow. Fore wing hyaline, venation yellowish brown. Metasoma and ovipositor sheaths brown.

***Sculpture*.** Head reticulate; clypeus radially striate. Mesosoma reticulate; propodeum finely reticulate. Metasoma weakly alutaceous and shiny.

***Head*.** Head in dorsal view 2.26 × as broad as long and 1.21 × as broad as mesoscutum; in frontal view 1.13 × as broad as high. Vertex with two distinct crests not composed of small sharp teeth, leaving a depression in middle. POL 1.45 × as long as OOL. Eye height 1.27 × eye length and 1.75 × as long as malar space. Distance between antennal toruli and lower margin of clypeus 1.36 × distance between antennal toruli and median ocellus. Antenna with scape 0.80 × as long as eye height and 1.03 × as long as eye length; pedicel 1.88 × as long as broad; combined length of pedicel and flagellum 1.18 × breadth of head; F1–F6 longer than broad, with 1 row of sensilla; clava 3.50 × as long as broad, with small micropilose area on each C3 and C4. Clypeal margin with one tooth.

***Mesosoma*.** Mesosoma 1.46 × as long as broad. Scutellum moderately depressed, 1.00 × as long as broad, frenal area indistinct. Propodeum 0.34 × as long as scutellum; nucha short. Fore wing 2.02 × as long as its maximum width; basal cell with several hairs; basal vein pilose; speculum partly closed below; M 0.75 × as long as PM and 1.50 × as long as S; stigma enlarged.

***Metasoma*.** Metasoma 1.40 × as long as broad, 1.00 × as long as mesosoma. Petiole strongly transverse. Ovipositor sheath projecting slightly beyond apex of metasoma.

**Male.** Unknown.

#### Etymology.

From the Latin *grandis* and *stigma*, referring to the wide stigma of fore wing of this species.

**Figures 48–55.**
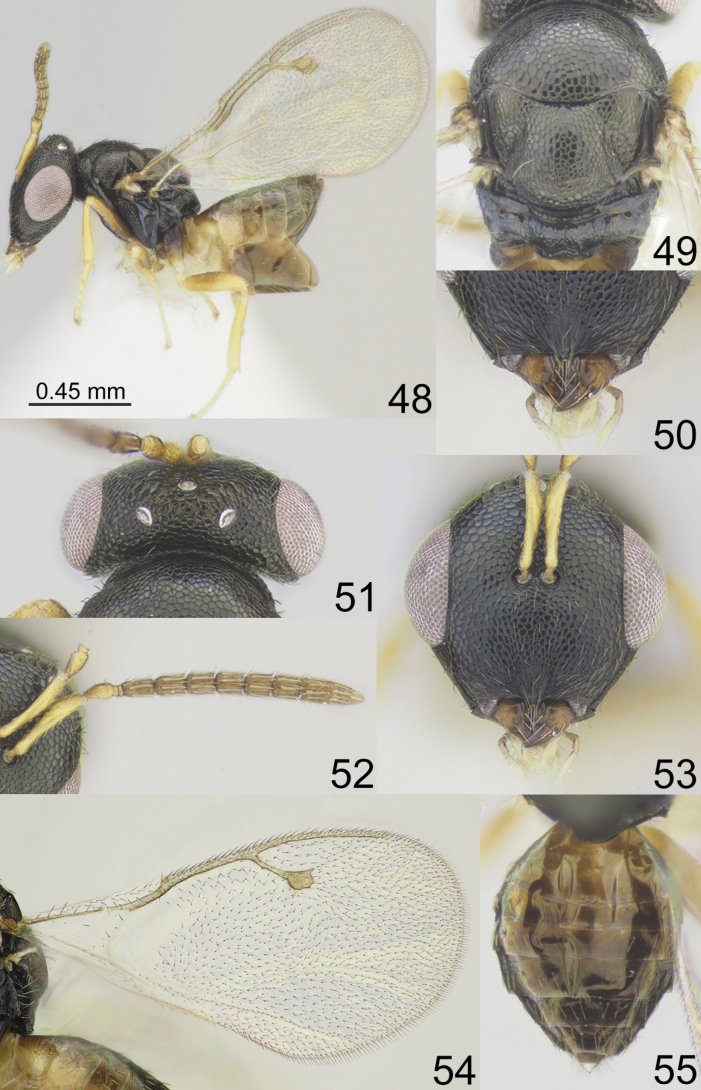
*Apsiloceragrandistigma* sp. nov., female, holotype **48** habitus, lateral view **49** mesosoma, dorsal view **50** clypeus **51** head and pronotum, dorsal view **52** antenna **53** head, frontal view **54** fore wing **55** metasoma, dorsal view.

#### Distribution.

Korean Peninsula.

#### Comments.

*Apsiloceragrandistigma* sp. nov. belongs to a group of species that have a vertex with two distinct crests not composed of small sharp teeth, leaving a depression in middle. This species is very similar to *A.bicristata*; the differences between these species are given in the key.

### ﻿ Apsilocera jejuensis

Taxon classificationAnimaliaHymenopteraPteromalidae

Tselikh, Lee & Ku sp. nov.

672B5BB0-6A9C-5C1B-A059-1C2D580A6F10

https://zoobank.org/55F61397-C5FE-41F7-8A22-B90FA6F3B6F9

Figs 56–63

#### Type material.

***Holotype*** • female, Republic of Korea: “Jeju-do, Jeju-si, Hanllim-eup, Geumak-ri, 16.VII–13.X.2021, Malaise trap, coll. Y.H. Park, M.H. Kim, D.H. Park, J.Y. Kim” (NIBR). ***Paratype*** • 1 female, “Jeju-do, Jeju-si, Jocheon-eup, Gyorae Nat. Recreat., forest, 33°26'28"N, 126°39'53"E, 13.VII.2023, coll. E. Tselikh” (ZISP).

#### Description.

**Female.** Body length 1.50–1.55 mm; fore wing length 1.43–1.50 mm.

***Coloration*.** Head black in dorsal view and dark blue-green with metallic diffuse luster in frontal view; antenna with scape yellowish brown; pedicel, anelli, F1–F6, and clava brown. Mesosoma black, but propodeum dorsally dark blue-green with metallic diffuse luster; fore and mid coxa black, hind coxae basally black, apically yellowish brown, all femora yellowish brown, all tibiae and tarsi yellow. Fore wing hyaline, venation yellowish brown. Metasoma dark brown with metallic green, diffuse coppery luster; ovipositor sheaths dark brown.

***Sculpture*.** Head finely reticulate; clypeus radially striate, but near clypeal margin smooth. Mesosoma finely reticulate; propodeum weakly alutaceous. Metasoma Mt2 and Mt3 smooth, Mt4–Mt8 weakly alutaceous and shiny.

***Head*.** Head in dorsal view 2.30–2.36 × as broad as long and 1.31–1.33 × as broad as mesoscutum; in frontal view 0.90–1.11 × as broad as high. Vertex with one continuous crest composed of small sharp teeth, crest height 0.50 × eye length. POL 0.85–0.87 × as long as OOL. Eye height 1.37–1.40 × eye length and 1.95–2.10 × as long as malar space. Distance between antennal toruli and lower margin of clypeus 4.40–4.15 × distance between antennal toruli and median ocellus. Antenna with scape 1.09–1.12 × as long as eye height and 1.44–1.53 × as long as eye length; pedicel 1.35–1.45 × as long as broad; combined length of pedicel and flagellum 0.95–0.98 × breadth of head; F1–F6 longer than broad, with 1 row of sensilla; clava 3.70–3.84 × as long as broad, with small micropilose areas on each C3 and C4. Clypeal margin rounded.

***Mesosoma*.** Mesosoma 1.30–1.36 × as long as broad. Scutellum moderately depressed, 0.77–0.81 × as long as broad, frenal area indistinct. Propodeum 0.45–0.48 × as long as scutellum; nucha short. Fore wing 2.24–2.28 × as long as its maximum width; basal cell with several hairs; basal vein pilose; speculum open below; M 0.90 × as long as PM and 2.10–2.22 × as long as S; stigma small.

***Metasoma*.** Metasoma 1.40–1.45 × as long as broad, 1.20–1.25 × as long as mesosoma. Petiole strongly transverse. Ovipositor sheath projecting slightly beyond apex of metasoma.

**Male.** Unknown.

#### Etymology.

The species is named in honor of the type locality, Jeju Island.

**Figures 56–63.**
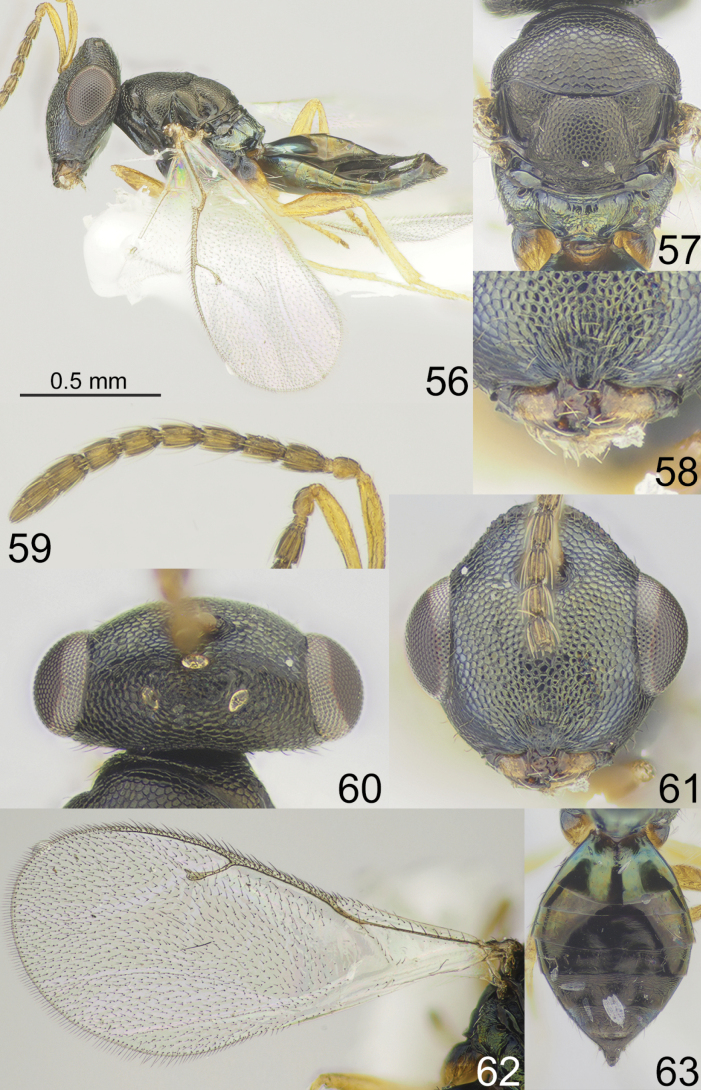
*Apsilocerajejuensis* sp. nov., female, holotype **56** habitus, lateral view **57** mesosoma, dorsal view **58** clypeus **59** antenna **60** head and pronotum, dorsal view **61** head, frontal view **62** fore wing **63** metasoma, dorsal view.

#### Distribution.

Korean Peninsula.

#### Comments.

*Apsilocerajejuensis* sp. nov. belongs to a group of species that have a vertex with one continuous crest composed of small sharp teeth. This species is very similar to *A.budai* sp. nov.; the differences between these species are given in the key.

### ﻿ Apsilocera marina

Taxon classificationAnimaliaHymenopteraPteromalidae

Tselikh, Lee & Ku sp. nov.

C54EF1A4-9705-5FC7-A52C-B6FD09E12E83

https://zoobank.org/0C2D724C-E018-49FE-9ABB-B80F8B51DA23

Figs 64–72

#### Type material.

***Holotype*** • female, Republic of Korea: “Jeollanam-do, Goheung-gun, Bongnae-myeon, Oenarodo, Malaise trap, 16–29.VIII.2020, coll. D.S. Ku, J.H. Lee” (NIBR). ***Paratype*** • 1 female, “Jeju-do, Jeju-si, Hanllim-eup, Geumak-ri, 16.VI–14.VII.2021, Malaise trap, coll. Y.H. Park, M.H. Kim, D.H. Park, J.Y. Kim” (ZISP).

#### Description.

**Female.** Body length 1.30–1.35 mm; fore wing length 1.40–1.45 mm.

***Coloration*.** Head black in dorsal view and dark blue in frontal view; antenna with scape yellowish brown; pedicel, anelli, F1–F6, and clava brown. Mesosoma black, but propodeum dorsally dark blue-green with metallic diffuse luster; fore and hind coxa black with metallic diffuse violet luster, mid coxae yellowish brown, all femora yellowish brown, all tibiae and tarsi yellow. Fore wing hyaline, venation yellowish brown. Metasoma dark brown with metallic green, diffuse coppery luster, laterally and ventrally brown; ovipositor sheaths dark brown.

***Sculpture*.** Head finely reticulate; clypeus radially striate. Mesosoma reticulate; propodeum alutaceous. Metasoma smooth and shiny.

***Head*.** Head in dorsal view 2.36–2.43 × as broad as long and 1.31–1.34 × as broad as mesoscutum; in frontal view 1.36–1.38 × as broad as high. Vertex with a row of teeth originating near eye and ending near posterior ocellus. POL 1.20–1.33 × as long as OOL. Eye height 1.31–1.35 × eye length and 1.60–1.67 × as long as malar space. Distance between antennal toruli and lower margin of clypeus 3.68–3.70 × distance between antennal toruli and median ocellus. Antenna with scape 1.00 × as long as eye height and 1.30–1.33 × as long as eye length; pedicel 1.48–1.50 × as long as broad; combined length of pedicel and flagellum 1.00 × breadth of head; F1–F6 longer than broad, with single row of sensilla; clava 2.60–2.85 × as long as broad, with small micropilose area on each C3 and C4. Clypeal margin with two teeth. Mandible formula 4:4.

***Mesosoma*.** Mesosoma 1.24–1.26 × as long as broad. Scutellum arched, 0.86–0.88 × as long as broad, frenal area indistinct. Propodeum 0.44 × as long as scutellum; nucha short. Fore wing 2.10–2.22 × as long as its maximum width; basal cell with two or three hairs; basal vein pilose; speculum partly open; M 0.85–0.87 × as long as PM and 1.80–1.86 × as long as S; stigma moderate enlarged.

***Metasoma*.** Metasoma 1.03–1.20 × as long as broad, 0.82–0.88 × as long as mesosoma. Petiole strongly transverse. Ovipositor sheath projecting slightly beyond apex of metasoma.

**Male.** Unknown.

**Figures 64–72.**
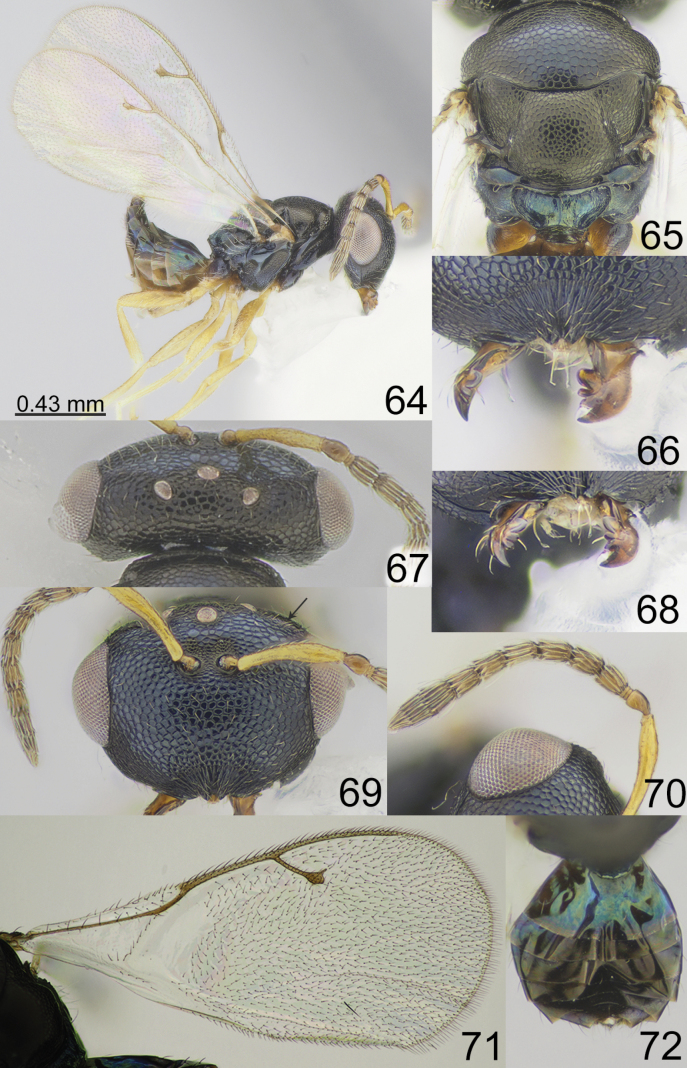
*Apsiloceramarina* sp. nov., female, holotype **64** habitus, lateral view **65** mesosoma, dorsal view **66** clypeus **67** head and pronotum, dorsal view **68** mandible **69** head, frontal view **70** antenna **71** fore wing **72** metasoma, dorsal view.

#### Etymology.

The species is named in honor of the senior author’s sister, Marina (Tselikh) Kopylova.

#### Distribution.

Korean Peninsula.

#### Comments.

*Apsiloceramarina* sp. nov. belongs to a group of species that have a vertex with a row of teeth originating near eye and ending near posterior ocellus. This species is very similar to *A.tuberculata* Mitroiu & Achterberg; the differences between these species are given in the key.

### ﻿ Apsilocera totoroi

Taxon classificationAnimaliaHymenopteraPteromalidae

Tselikh, Haas & Ku sp. nov.

C064F5AD-8057-5023-B71B-4C91EC07627D

https://zoobank.org/5E49E91F-0A53-4490-A810-EEECB758FA84

Figs 73–80

#### Type material.

***Holotype*** • female, Japan: “Honshu, Fukushima Pref., HinoemataVill., 16–18.VIII.1999, coll. S. Belokobylskij” (ZISP). ***Paratypes*** • 1 female (SMNS_Hym_T00799), “Sweden, Mörbylånga, Kalkstad NR (#136/2014), mixed deciduous forest, 31.VII–27.VIII.2014, Malaise trap, 56.609569N, 16.503726E, coll. M. Jaschhof, C. Jaschhof”, Sample ID: SMNS_47998 (SMNS) • 2 females (SMNS_Hym_T00800 & SMNS_Hym_T00801), “Germany, Baden-Württemberg, Lkr. Rems-Murr-Kreis, Aspach bei Backnang, Forest, 15.VII–8.VIII.2013, Malaise trap, 48.998972N, 9.419746E, leg. L. Krogmann, J. Holstein, T. Kothe”, Sample ID: SMNS_50181 & SMNS_50214 (SMNS).

#### Description.

**Female.** Body length 1.50–1.99 mm; fore wing length 1.36–1.76 mm.

***Coloration*.** Head black; antenna with scape, pedicel, and anelli yellowish brown, F1–F6 and clava brown. Mesosoma black, but propodeum dorsally dark blue with metallic diffuse luster. Fore coxae black or dark brown, mid and hind coxae yellowish brown; all femora and tibiae yellowish brown, all tarsi yellow. Fore wing hyaline, venation yellowish brown. Metasoma dorsally dark brown, but Mt2 dorsally dark metallic blue-green; laterally and ventrally brown; ovipositor sheaths brown.

***Sculpture*.** Head reticulate; clypeus radially striate. Mesosoma with propodeum reticulate. Metasoma weakly alutaceous and shiny.

***Head*.** Head in dorsal view 2.33–2.53 × as broad as long and 1.23–1.37 × as broad as mesoscutum; in frontal view 1.40–1.67 × as broad as high. Vertex with regular sculpture. POL 1.13–1.29 × as long as OOL. Eye height 1.24–1.38 × eye length and 1.80–1.95 × as long as malar space. Distance between antennal toruli and lower margin of clypeus 1.92–2.10 × distance between antennal toruli and median ocellus. Antenna with scape 0.96–1.07 × as long as eye height and 1.30–1.38 × as long as eye length; pedicel 1.50–1.67 × as long as broad; combined length of pedicel and flagellum 0.91–1.10 × breadth of head; F1–F6 longer than broad, with a row of sensilla; clava 3.00–3.40 × as long as broad, with small micropilose area on each C3 and C4. Clypeal margin with two small teeth. Mandible formula 4:4.

***Mesosoma*.** Mesosoma 1.29–1.48 × as long as broad. Scutellum moderately depressed, 0.92 × as long as broad, frenal area indistinct. Propodeum 0.44–0.53 × as long as scutellum. Fore wing 1.96–2.12 × as long as its maximum width; basal cell bare; basal cell with 0–4 hairs; basal vein pilose; speculum partly closed below; M 0.96–1.00 × as long as PM and 2.27–2.33 × as long as S, stigma small.

**Figures 73–80.**
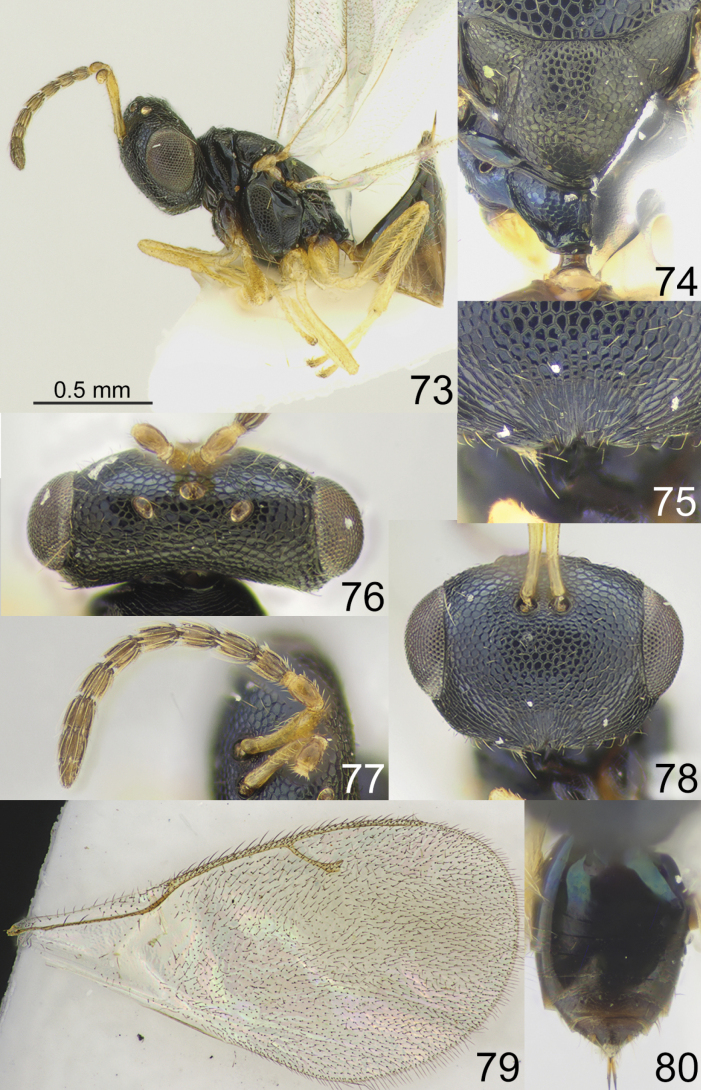
*Apsiloceratotoroi* sp. nov., female, holotype **73** habitus, lateral view **74** scutellum and propodeum, dorsal view **75** clypeus **76** head, dorsal view **77** antenna **78** head, frontal view **79** fore wing **80** metasoma, dorsal view.

***Metasoma*.** Metasoma 1.45–1.82 × as long as broad and 0.85–1.28 × as long as mesosoma. Petiole strongly transverse. Ovipositor sheath projecting slightly beyond apex of metasoma.

**Male.** Unknown.

#### Etymology.

The species is named in honor of the “My Neighbor Totoro” character – “Totoro” of Hayao Miyazaki.

#### Distribution.

Germany, Japan, Sweden.

#### Comments.

*Apsiloceratotoroi* sp. nov. belongs to a group of species that have a vertex with regular sculpture. This species is very similar to *A.bramleyi* and *A.triapitzini* sp. nov.; the differences between these species are given in the key.

### ﻿ Apsilocera triapitzini

Taxon classificationAnimaliaHymenopteraPteromalidae

Tselikh, Haas & Ku sp. nov.

3DAD7C09-EFEB-56FC-BB25-028FCFA70628

https://zoobank.org/DB674A4F-0616-4300-A2D9-42BA3BF5547E

Figs 81–88 , 98

#### Type material.

***Holotype*** • female, Republic of Korea: “Gyeongsangnam-do, Goseong-gun, Hail-myeon, Suyang-ri, 34°58'34.8"N, 128°12'08.3"E, 18.VI.2022, coll. E. Tselikh” (NIBR). ***Paratypes*** • 1 male, same labels as holotype (SMNE) • 1 female, “Gyeongsangnam-do, Geochang-gun, Mari-myeon, Yeongseung-ri, 35.714060N, 127.876007E, 06.VII.2023, coll. E. Tselikh” (ZISP) • 1 female, “Russia, Primirskii Reg., Lazovsky Reserve, Preobrazhenie Vill., 18–19.VIII.2010, coll. E. Tselikh” (ZISP) • 1 female (SMNS_Hym_T00798), “Sweden, Mörbylånga, Kalkstad (#114/2014), mixed deciduous forest, 27.VI–30.VII.2014, Malaise trap, 56.609569N, 16.503726E, coll. M. Jaschhof, C. Jaschhof”, Sample ID: 48265 (SMNS) • 1 female (NHRS-HEVA000022838), “Sweden Öl, Mörbylånga kommun, Västerstads almlunds naturreservat., Old elm forest (3002 Trap ID, 3053 Event ID), 10.VI-9.VII.2014, Malaise trap, 56.427648N, 16.421110E, leg. Swedish Malaise Trap Project”, Sample ID: SMNS_50682 (NRM).

#### Description.

**Female.** Body length 1.20–1.61 mm; fore wing length 1.05–1.36 mm.

***Coloration*.** Head black; antenna with scape yellowish brown; pedicel, anelli, F1–F6, and clava brown. Mesosoma black, but propodeum dorsally dark blue with metallic diffuse luster. Fore coxae metallic blue, mid coxae yellowish brown, hind coxae basally metallic blue, apically yellowish brown; all femora and tibiae yellowish brown, all tarsi yellow. Fore wing hyaline, venation yellowish brown. Metasoma dorsally brown with metallic green diffuse coppery luster, laterally and ventrally yellowish brown; ovipositor sheaths brown.

***Sculpture*.** Head grossly reticulate; clypeus radially striate, but near clypeal margin smooth. Mesosoma grossly reticulate; propodeum alutaceous. Metasoma weakly alutaceous and shiny.

***Head*.** Head in dorsal view 2.15–2.32 × as broad as long and 1.24–1.34 × as broad as mesoscutum; in frontal view 1.37–1.52 × as broad as high. POL 1.22–1.50 × as long as OOL. Eye height 1.24–1.33 × eye length and 1.94–2.05 × as long as malar space. Distance between antennal toruli and lower margin of clypeus 1.87–2.21 × distance between antennal toruli and median ocellus. Antenna with scape 0.90–0.97 × as long as eye height and 1.12–1.28 × as long as eye length; pedicel 1.33–1.45 × as long as broad; combined length of pedicel and flagellum 0.99–1.15 × breadth of head; F1–F4 longer than broad, F5, F6 subsquare, with single row of sensilla; clava 2.70–3.23 × as long as broad, with small micropilose area on each C3 and C4. Clypeal margin produced and emarginate medially. Mandible formula 3:4.

**Figures 81–88.**
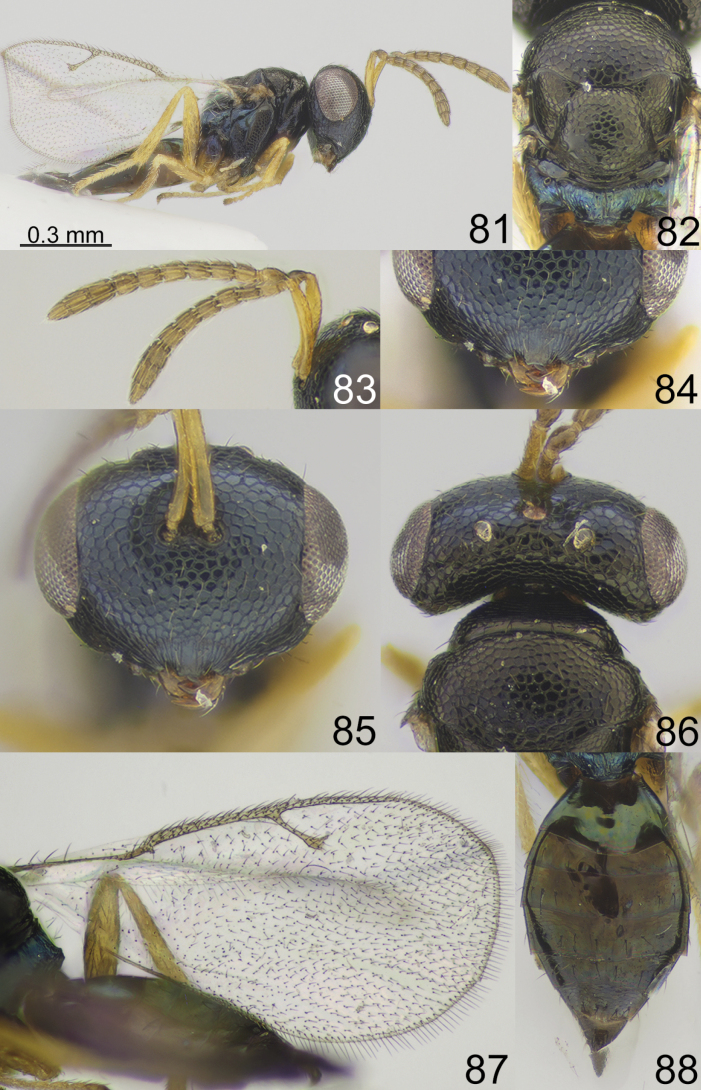
*Apsiloceratriapitzini* sp. nov., female, holotype **81** habitus, lateral view **82** mesosoma, dorsal view **83** antenna **84** clypeus **85** head, frontal view **86** head and pronotum, dorsal view **87** fore wing **88** metasoma, dorsal view.

***Mesosoma*.** Mesosoma 1.30–1.42 × as long as broad. Scutellum arched, 0.77 × as long as broad, frenal area indistinct. Propodeum 0.39–0.50 × as long as scutellum. Fore wing 2.02–2.09 × as long as its maximum width; basal cell bare; basal vein pilose; speculum partly closed below; M 0.93–1.13 × as long as PM and 2.20–2.38 × as long as S, stigma small.

***Metasoma*.** Metasoma 1.53–1.77 × as long as broad and 1.22–1.35 × as long as mesosoma. Petiole strongly transverse. Ovipositor sheath projecting slightly beyond apex of metasoma.

**Male.** Body length 0.95 mm; fore wing length 0.90 mm. Eye height 2.46 × as long as malar space. Distance between antennal toruli and lower margin of clypeus 3.16 × distance between antennal toruli and median ocellus. Antenna with pedicel 1.60 × as long as broad; clava 5.60 × as long as broad; combined length of pedicel and flagellum 1.29 × breadth of head. Metasoma 2.30 × as long as broad and 0.97 × as long as mesosoma. Otherwise, similar to female.

#### Etymology.

The species is named in honor of the prominent entomologist, an expert on Encyrtidae (Hymenoptera), Dr., Prof. Vladimir A. Trjapitzin.

#### Distribution.

Russia, Republic of Korea, Sweden.

#### Comments.

*Apsiloceratriapitzini* sp. nov. belongs to a group of species that have a vertex with regular sculpture. This species is very similar to *A.bramleyi*; the differences between these species are given in the key.

### ﻿ Apsilocera verticillata

Taxon classificationAnimaliaHymenopteraPteromalidae

Bouček, 1956

3451356E-2006-5FF5-82A9-AE3D3E484B92

Figs 89–96 , 97

Apsilocera verticillata Bouček, 1956: 319. Holotype male (NMPC, examined).

#### Type material.

***Holotype*** • male, Slovakia: “Slov. mer.: Gbelce 29.7.55 Bouček”, “Holotypus”, “Mus. Nat. Pragae Inv. 3086”, “*Apsiloceraverticillata* ♂ Bčk. Det Z. Bouček, 1955” (RMNH).

#### Additional material examined.

Japan • 1 female, 1 male, “Chiba Pref., Ichinomiya-Machi, 25.X.2002, coll. K. Kubo” (EIHU). Russia • 1 female, “Primorskii Reg., Spassk-Dal’niy Vill., 2–4.VII.2011, coll. S. Belokobylskij” (ZISP). Republic of Korea • 1 female, “Gyeongsangnam-do, Namhae-gun, Sangiu-myeon, Sangiu-ri, 13.VIII.2022, coll. D.S Ku, J.H. Lee, H.J. Jeong” (NIBR) • 1 female, “Gyeongsangnam-do, Geochang-gun, Namsang-myeon, Jeoncheok-ri, 35°37'15.3"N, 127°57'51.4"E, 22.VI. 2023, coll. S. Belokobylskij” (ZISP) • 5 females, “Gyeongsangbuk-do, JuWangSan-myeon, Cheongsong-gun, JuWangSan SW, 21.IV.2023, 3.V.2023, 31.V.2023, 18.VII.2023, coll. J.H. Lee (SMNE).

#### Description.

**Female.** Body length 1.18–1.25 mm; fore wing length 0.98–1.05 mm.

***Coloration*.** Head black; antenna with scape, pedicel, anelli, and F1–F6 yellowish brown, clava brow. Mesosoma black, but propodeum dorsally dark blue-green with metallic diffuse luster. All coxae yellowish brown; all femora, tibiae and tarsi yellow. Fore wing hyaline, venation yellowish brown. Metasoma dorsally brown, laterally and ventrally yellowish brown; ovipositor sheaths yellowish brown.

***Sculpture*.** Head reticulate; clypeus radially striate. Mesosoma reticulate; propodeum smooth and shiny. Metasoma smooth and shiny, but Mt7 weakly alutaceous and shiny.

***Head*.** Head in dorsal view 2.55–2.60 × as broad as long and 1.37–1.49 × as broad as mesoscutum; in frontal view 1.48–1.50 × as broad as high. Vertex with regular sculpture. POL 1.20–1.30 × as long as OOL. Eye height 1.34–1.36 × eye length and 2.10–2.36 × as long as malar space. Distance between antennal toruli and lower margin of clypeus 1.98–2.00 × distance between antennal toruli and median ocellus. Antenna with scape 0.90–0.95 × as long as eye height and 1.29–1.32 × as long as eye length; pedicel 1.40–1.46 × as long as broad; combined length of pedicel and flagellum 0.90–0.92 × breadth of head; F1–F6 longer than broad with a row of sensilla; clava 2.89–3.30 × as long as broad, with small micropilose area on each C3 and C4. Clypeal with one small tooth. Mandible formula 3:4.

***Mesosoma*.** Mesosoma 1.30–1.32 × as long as broad. Scutellum moderately depressed, 0.70–0.73 × as long as broad, frenal area indistinct. Propodeum 0.51–0.54 × as long as scutellum. Fore wing 2.07–2.12 × as long as its maximum width; basal cell bare; basal vein pilose; speculum partly closed below; M 1.03–1.17 × as long as PM and 2.10–2.20 × as long as S, stigma small.

***Metasoma*.** Metasoma 1.41–1.43 × as long as broad and 1.00–1.04 × as long as mesosoma. Petiole strongly transverse. Ovipositor sheath projecting slightly beyond apex of metasoma.

**Male.** Body length 1.25–1.30 mm; fore wing length 1.10–1.15 mm. Head in dorsal view 2.30–2.35 × as broad as long. POL 1.40–1.55 × as long as OOL. Combined length of pedicel and flagellum 1.30–1.35 × breadth of head. Metasoma 1.65–1.73 × as long as broad. Otherwise, similar to female.

#### Distribution.

Czech Republic, Japan, Moldova, Netherlands, Republic of Korea, Russia, Slovakia, Sweden.

#### Comments.

*Apsiloceraverticillata* Bouček belongs to a group of species that have a vertex with regular sculpture. This species is very similar to *A.maculata* Mitroiu & Achterberg; the differences between these species are given in the key.

## Supplementary Material

XML Treatment for Apsilocera

XML Treatment for Apsilocera bradburyi

XML Treatment for Apsilocera bramleyi

XML Treatment for Apsilocera budai

XML Treatment for Apsilocera dupla

XML Treatment for Apsilocera eleganta

XML Treatment for Apsilocera elongata

XML Treatment for Apsilocera grandistigma

XML Treatment for Apsilocera jejuensis

XML Treatment for Apsilocera marina

XML Treatment for Apsilocera totoroi

XML Treatment for Apsilocera triapitzini

XML Treatment for Apsilocera verticillata

## ﻿Additional information

### Author ORCIDs

Ekaterina V. Tselikh https://orcid.org/0000-0002-9184-043X

Jaehyeon Lee https://orcid.org/0000-0002-3666-6029

Michael Haas https://orcid.org/0000-0001-6869-6698

Mircea-Dan Mitroiu https://orcid.org/0000-0003-1368-7721

Deok-Seo Ku https://orcid.org/0000-0002-6274-6479

### Data availability

All of the data that support the findings of this study are available in the main text.
